# Elevated emotional contagion in a mouse model of Alzheimer’s disease is associated with increased synchronization in the insula and amygdala

**DOI:** 10.1038/srep46262

**Published:** 2017-04-07

**Authors:** Jiye Choi, Yong Jeong

**Affiliations:** 1 Department of Bio and Brain Engineering, Korea Advanced Institute of Science and Technology (KAIST), Daejeon, 34141, Republic of Korea; 2 KI Health Science and Technology, Korea Advanced Institute of Science and Technology (KAIST), Daejeon, 34141, Republic of Korea

## Abstract

Emotional contagion, a primitive form of empathy, is heightened in patients with Alzheimer’s disease (AD); however, the mechanism underlying this attribute has not been thoroughly elucidated. In this study, observational fear conditioning was performed to measure emotional contagion levels in a mouse model of AD. Simultaneous recording of local field potentials in the bilateral anterior insula, basolateral amygdala, anterior cingulate cortex, and retrosplenial cortex was also conducted to investigate related brain network changes. Consistent with the results obtained with AD patients, 11-month-old AD model mice exhibited significantly higher freezing levels in observational fear conditioning, indicating elevated emotional contagion compared to age-matched wild-type mice. Furthermore, the left anterior insula and right basolateral amygdala of 11-months-old AD model mice indicated sustained increases in synchronization when they observed the suffering of conspecifics. These changes did not appear in other age groups or wild-type controls. Additionally, the amyloid plaque burden within the anterior insula was significantly correlated with the freezing levels in observational fear conditioning. Taken together, this study reveals increased and sustained network synchrony between the anterior insula and basolateral amygdala, which comprise a salience network in humans, as a potential mechanism for elevated emotional contagion in a mouse model of AD.

Emotional symptoms, in addition to cognitive symptoms^1^, have been frequently observed in patients with Alzheimer’s disease (AD)^2^. Although cognitive deficits such as memory impairment are regarded as typical symptoms of AD, emotional or neuropsychiatric symptoms are often considered more difficult problems, as these symptoms can be distressing and burdensome to caregivers^3^,^4^. Patients commonly show emotional alterations such as depression^5^,^6^, aggression^7^, and anxiety^8^, which have also been verified in studies using animal models of AD^9^,^10^,^11^,^12^. Recently, resting-state functional magnetic resonance imaging studies of patients with AD have suggested that increased connectivity in the salience network (SN), which includes regions such as the anterior cingulate cortex and anterior insula^13^, is related to the emotional changes associated with hyperactivity symptoms^14^,^15^. It is likely that these symptoms are typically related to the connectivity of multiple brain regions rather than to a deficit in one particular area^16^. However, few animal model studies have investigated the emotional symptoms of AD at the network level.

One recent study reported that patients with AD showed heightened emotional contagion, which is the ability to unconsciously mimic the emotions of others^17^. It has been suggested that a higher level of emotional contagion contributes to a greater sensitivity to the mood and behaviours of surrounding people^18^. However, the causal mechanism of this symptom has not yet been fully identified.

As emotional contagion is not only observed in humans but also in rodents^19^,^20^, this behaviour is regarded as a primitive form of empathy. In the present study, observational fear conditioning (OFC), a method that measures the level of emotional contagion in mice^21^, was performed to determine changes in emotional contagion levels in a mouse model of AD. To test our hypothesis that SN-like regions are responsible for elevated emotional contagion, local field potentials (LFPs) and amyloid burden were measured in the SN-like regions of a mouse model of AD and analysed in relation to OFC behaviour.

## Results

### OFC freezing level is increased in the AD model mice at 11 months of age

**Figure 1:**
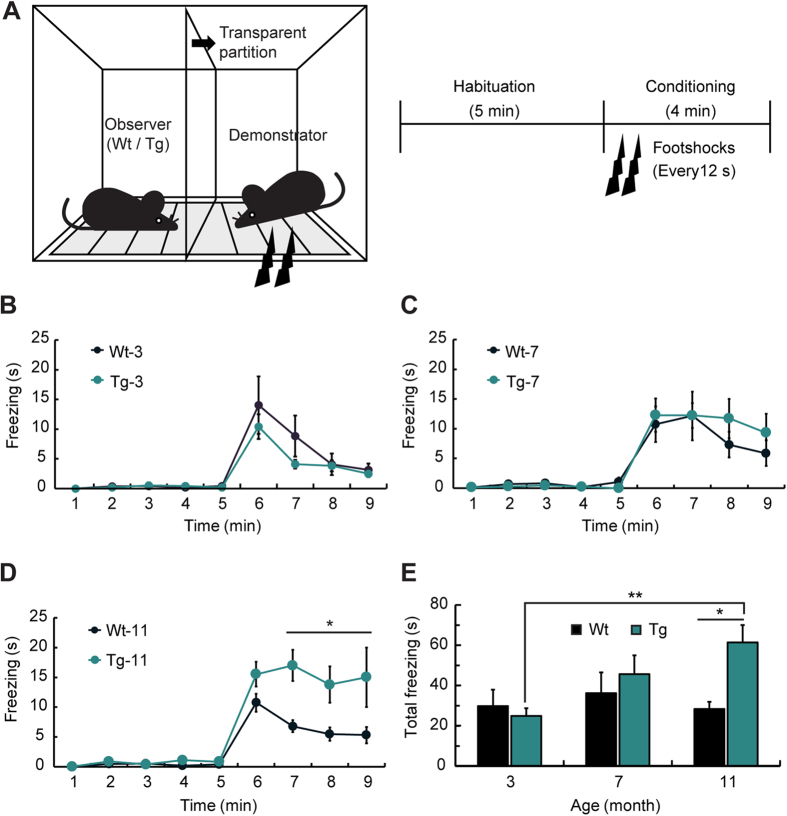
Tg-11 mice showed elevated fear response on the suffering of conspecific. (**A**) Observational fear conditioning (OFC) apparatus and the experimental procedure of OFC. (**B–D)** Freezing levels during OFC in Wt and Tg mice at 3 months (Wt-3: n = 8; Tg-3: n = 9) (**B**), 7 months (Wt-7: n = 10; Tg-7: n = 8) (**C**), and 11 months of age (Wt-11: n = 9; Tg-11: n = 8) (**D**). (**B,C**) Tg-3 and Tg-7 mice did not show any difference in the freezing duration compared to the Wt littermates. (**D**) Tg-11 mice showed significantly higher freezing levels than Wt-11 mice during the conditioning period (*P* < 0.005 (genotype), two-way repeated analysis of variance (ANOVA), *P < 0.05, Šidák’s *post hoc* test). (**E**) Total freezing duration of all age groups in the 4 min conditioning session. Tg-11 mice showed significantly higher total freezing than in Tg-3 and in Wt-11 mice (*P* < 0.05 (age*genotype interaction), *P* < 0.05 (age factor), two-way ANOVA, *P < 0.05, **P < 0.01, Bonferroni’s *post hoc* test). Data are presented as the mean ± SEM.

To examine the level of emotional contagion in a mouse model of AD, we performed the OFC test, which was previously reported as a method for measuring the fear response induced by the suffering of others^21^. Three-, seven-, and eleven-month-old male APPSWE/PS1E9 transgenic (Tg-3, Tg-7, and Tg-11, respectively) mice and their age-matched wild-type (Wt-3, Wt-7, and Wt-11, respectively) littermates were used. As the test apparatus was separated by a transparent partition, the observer mouse could watch the demonstrator’s pain response elicited through foot shocks (Fig. 1A). The freezing response of the observer (Wt or Tg mice) appeared in the conditioning session but rarely in the habituation session. Tg-3 and Tg-7 mice did not show any difference in freezing duration compared to the Wt littermates, whereas Tg-11 mice showed significantly higher freezing levels than those of Wt-11 mice (Fig. 1B–D; *P < 0.05). Total freezing during the conditioning session was significantly higher in Tg-11 mice than in Wt-11 mice (Fig. 1E; *P < 0.05), even though there was no locomotor activity difference between the two groups (Supplementary Fig. S1A). Furthermore, the individual OFC freezing level of Tg-11 mice was not correlated with locomotion in the open field test (Supplementary Fig. S1B; *r* = – 0.024, *P* = 0.954). The freezing duration in the contextual fear-conditioning test, however, was significantly less in Tg-11 mice than Wt-11 mice (Supplementary Fig. S1C; *P < 0.05), indicating fear memory impairment, which was not correlated with freezing levels during OFC (Supplementary Fig. S1D; *r* = 0.121, *P* = 0.775). Because there was no difference in the freezing levels in the demonstrators of each group (Supplementary Fig. S2A), this result did not reflect freezing level changes in the demonstrators. Moreover, the number of faecal droppings, an indirect measure of fear, was also significantly increased in Tg-11 mice compared to Wt-11 mice during the OFC test (Supplementary Fig. S2B; *P < 0.05).

### Synchronized theta oscillation between the left AI and right BLA increased most significantly during OFC in Tg mice

**Figure 2:**
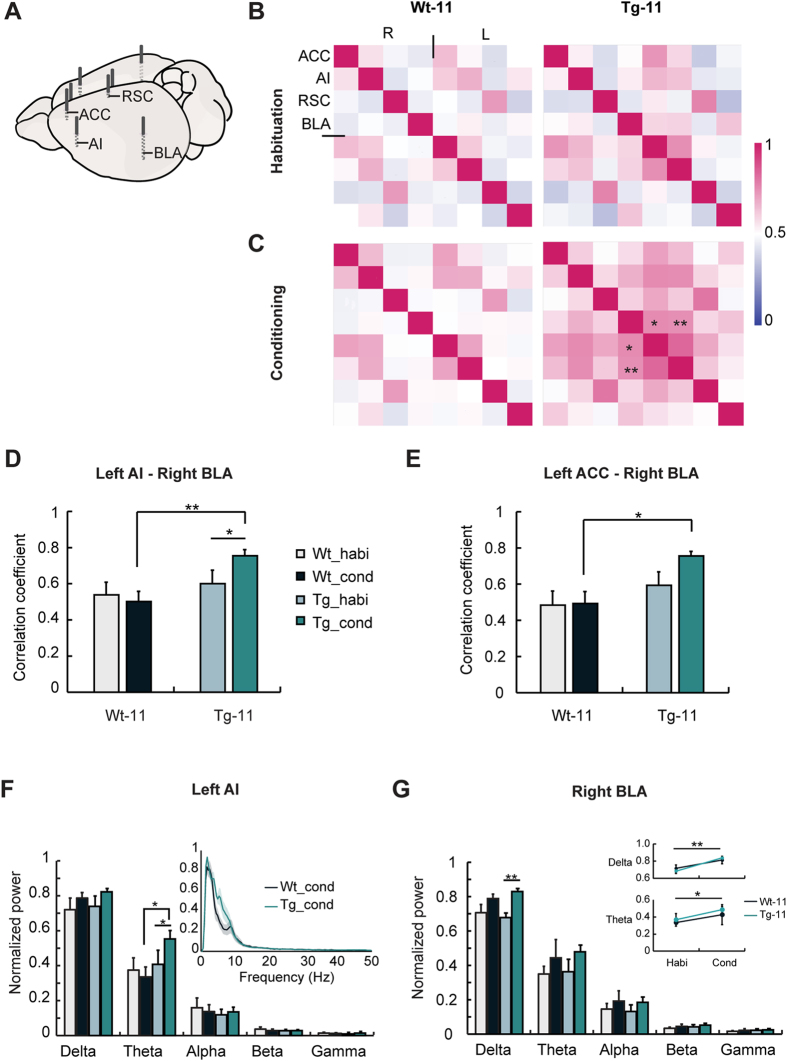
Left AI and right BLA in Tg-11 mice showed significantly higher cross-correlation during the conditioning period. (**A**) For LFP recording, eight electrodes were implanted to each region bilaterally (ACC: anterior cingulate cortex; AI: anterior insula; RSC: retrosplenial cortex; BLA: basolateral amygdala). (**B,C**) Cross-correlation matrix of Wt-11 (n = 7) and Tg-11 (n = 10) mice during the habituation (**B**) and conditioning periods (**C**). *P < 0.05, **P < 0.01 compared to Wt subjects. Two-way ANOVA and Bonferroni’s *post hoc* test were used. See Fig. 2D and E. (**D,E**) Bar graphs for cross-correlation coefficient. (**D**) Cross-correlation between the left AI and right BLA in Wt-11 and Tg-11 mice. During the conditioning session, the correlation between the two regions was significantly higher in Tg-11 mice than in Wt-11 mice (*P* < 0.05 (genotype*test interaction), *P* < 0.05 (genotype), two-way repeated ANOVA, **P < 0.01, Bonferroni’s *post hoc* test). The correlation in Tg-11 mice also showed a significant difference during the conditioning periods compared to the habituation (*P < 0.05, Bonferroni’s *post hoc* test). (**E**) Cross-correlation between the left ACC and right BLA in Wt-11 and Tg-11 mice. During the conditioning session, the correlation in Tg-11 mice was significantly higher than in Wt-11 mice (*P* < 0.05 (genotype), two-way repeated ANOVA, *P < 0.05, Bonferroni’s *post hoc* test). (**F,G**) Histogram of normalized LFP power of the left AI (**F**), right BLA (**G**). (**F**) Significant theta power increases appeared in the left AI of Tg-11 mice during the conditioning period compared to Wt-11 mice (*P* < 0.05 (genotype*test interaction), two-way repeated ANOVA, *P < 0.05, Bonferroni’s *post hoc* test). The theta power in Tg-11 mice also showed a significant increases during the conditioning periods compared to the habituation (*P < 0.05, Bonferroni’s *post hoc* test). Inset, Normalized power spectra of the left AI. (**G**) There was a significant difference in normalized delta power in the right BLA of Tg-11 mice during the conditioning period compared to the habituation (two-way repeated ANOVA, **P < 0.01, Bonferroni’s *post hoc* test). Inset, Differences between the habituation and conditioning periods in the delta and theta powers of the right BLA. Delta and theta powers were significantly increased during the conditioning periods compared to the habituation (**P < 0.01 (test) in delta power, *P < 0.05 (test) in theta power, two-way ANOVA). There was no difference in the right BLA power between two genotypes. habi: Habituation. cond: Conditioning. Data are presented as the mean ± SEM.

To investigate underlying brain network changes in Tg-11 mice exhibiting elevated emotional contagion, we recorded LFPs in the anterior cingulate cortex (ACC), anterior insula (AI), retrosplenial cortex (RSC), and basolateral amygdala (BLA) bilaterally during OFC (Fig. 2A). The locations of electrodes were verified after LFP recording (Supplementary Fig. S3A–D). Cross-correlation coefficients for each pair of theta oscillations were measured from eight regions of interest, and network matrices were generated based on these values (Fig. 2B,C). The LFPs were analysed for the habituation and conditioning periods of OFC, which represented the sessions without and with observations of pain response, respectively. The correlation between the left AI and right BLA in Tg-11 mice was significantly higher during the conditioning session compared to the habituation. During the conditioning periods, the correlation between these two regions was significantly higher in Tg-11 mice than in Wt-11 mice (Fig. 2C,D; *P < 0.05, **P < 0.01). In addition, the correlation between the left ACC and right BLA in Tg-11 mice was also significantly increased compared to Wt control mice (Fig. 2C,E; *P < 0.05). The correlation between the left AI and right BLA in Tg mice was significantly increased as the mice got older, whereas these differences did not appear in Wt mice group (Supplementary Fig. S4A,B; **P < 0.01). The correlation between the left ACC and right BLA in Wt and Tg mice also showed a similar tendency (Supplementary Fig. S4C,D; **P < 0.01).

Additionally, LFP power analyses of the habituation and conditioning sessions were conducted. The normalized theta power in the left AI of Tg-11 mice was significantly higher during the conditioning session compared to the habituation. The power in the left AI was significantly higher in Tg-11 mice than in Wt-11 mice during the conditioning period. (Fig. 2F; *P < 0.05). The normalized delta and theta powers in the right BLA were significantly higher during the conditioning session compared to the habituation (Fig. 2G; *P < 0.05, **P < 0.01). In the case of bilateral RSC regions, the normalized powers in delta range were much lower in Tg-11 mice than in Wt-11 mice (Supplementary Fig. S5A,B; *P < 0.05, ^#^*P* < 0.05). However, other LFP recording sites did not show any significant change (data not shown).

### Changes in network correlation are sustained while Tg mice observe the pain of demonstrators

**Figure 3:**
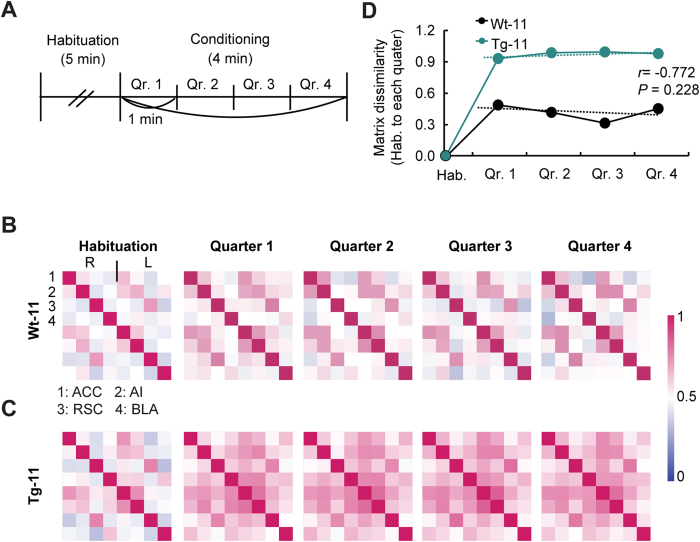
Changes in the network correlation of Tg-11 mice remained longer during the conditioning session in OFC. (**A**) Schematic drawing of the divided sessions for analysis. Qr: Quarter. (**B,C**) Cross-correlation matrix during the habituation and 4 quarters of Wt-11 and Tg-11 mice (Wt-11: n = 7; Tg-11: n = 10). (**D**) Matrix dissimilarity was calculated based on the Euclidean distance (see Methods) from the habituation matrix to each quarter matrix. The dotted lines represent the trend lines of the dynamics of each matrix. There was no significant relationship between the two groups (r = −0.772 *P* = 0.228, Pearson’s correlation).

To investigate changes of the correlation matrix over time, the conditioning period was divided into four quarters (Fig. 3A). During the first quarter, the matrices of both Wt-11 and Tg-11 mice displayed general increases in network correlation. However, whereas the changes of Tg mice increased in subsequent quarters (Fig. 3C), those of Wt mice returned to the initial state (Fig. 3B). To quantify this result, matrix dissimilarity from the habituation matrix to each quarter matrix was measured. The dissimilarity index of Tg mice remained elevated over time, whereas that of Wt mice decreased (Fig. 3D). There was no correlation between the matrix dynamics of the two groups (*r* = −0.772, *P* = 0.228).

### Amyloid burden in the AI of Tg mice showed the highest correlation with the OFC freezing levels

**Figure 4:**
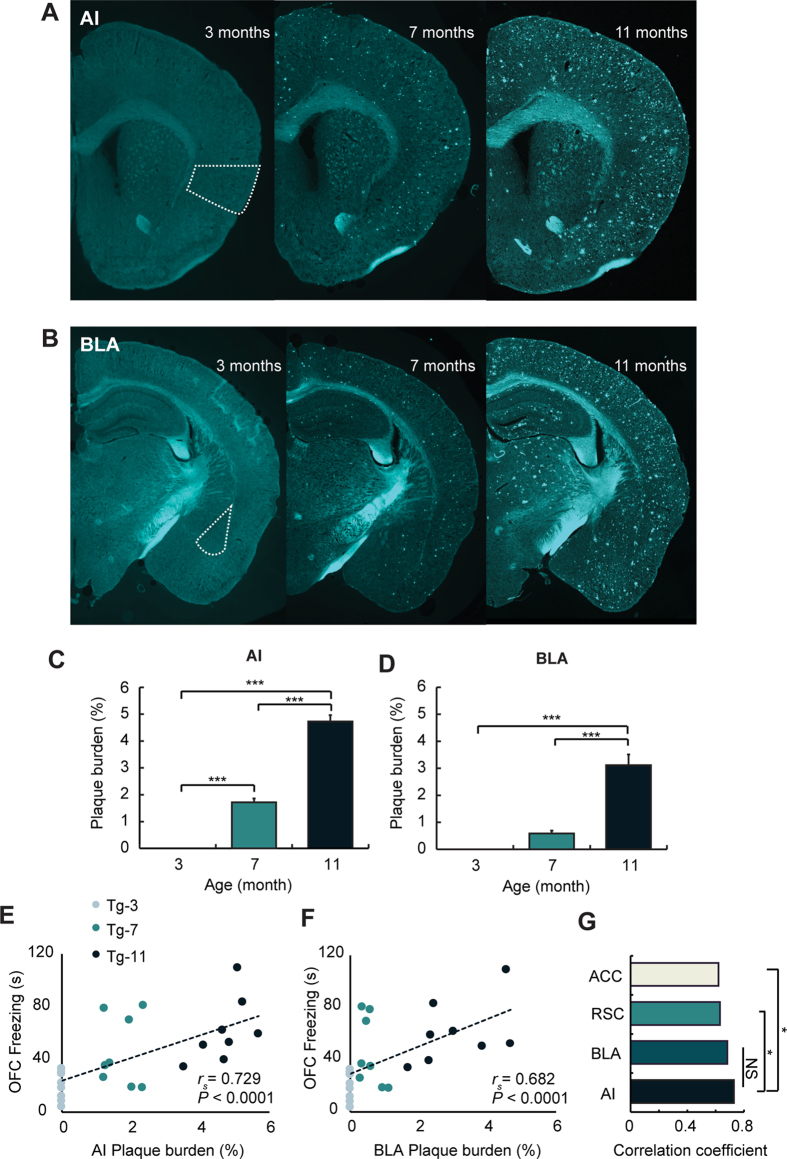
Plaque burden in the AI of Tg mice is highly correlated with the level of fear response in OFC. **(A,B)** Examples of amyloid plaque deposition in the AI (**A**) and BLA (**B**) of Tg-3, Tg-7, and Tg-11 mice. All brain slice samples were stained with thioflavin-S. Turquoise was used as a pseudo-colour for amyloid plaque. (**C,D**) Average plaque burden of the AI (**C**) and BLA (**D**) were quantified in Tg-3, Tg-7, and Tg-11 mice. (**C**) In the AI, all three age groups showed significant differences between each pair of groups (*P* < 0.0001 (age), one-way ANOVA, ***P < 0.001, Bonferroni’s *post hoc* test). (**D**) Plaque burden in the BLA of Tg-11 mice showed significantly higher than that of Tg-3 or Tg-7 mice (*P* < 0.0001 (age), one-way ANOVA, ***P < 0.001, Bonferroni’s *post hoc* test). (**E,F**) Plaque burden in the AI (**E**; *r*_*s*_ = 0.729, *P* < 0.0001) and BLA (**F**; *r*_*s*_ = 0.682, *P* < 0.0001) showed a significant correlation with the freezing levels in OFC. Spearman’s correlation was used for the significance test. (**G**) The correlation coefficient of the AI was significantly higher than that of the RSC or ACC (*P < 0.05, Steiger’s z-test). Data are presented as the mean ± SEM.

To identify correlations between the OFC freezing levels and the amyloid plaque burden, the plaque area was quantified at the AI, BLA, RSC, and ACC of all groups (Fig. 4A,B; Supplementary Fig. S6A,B). No accumulation was observed in Wt-3, Wt-7, and Wt-11 mice. Although amyloid plaques were not observed in Tg-3 mice, these structures appeared in Tg-7 mice and were significantly increased in Tg-11 mice at the AI (Fig. 4C; ***P < 0.001). In the BLA, there was no difference between Tg-3 and Tg-7 mice, but Tg-11 mice showed a significantly higher plaque burden compared with Tg-3 or Tg-7 animals (Fig. 4D; ***P < 0.001). The RSC and ACC also showed significant increases in plaque burden with increasing age of the Tg mice (Supplementary Fig. S6C,D). The plaque burden in the AI showed the highest correlation with OFC freezing levels (Fig. 4E; *r*_*s*_ = 0.729, *P* < 0.0001). Other regions also showed significant correlations with OFC freezing levels (Fig. 4F; *P* < 0.0001; Supplementary Fig. S6E,F; *P* < 0.001), but the correlation coefficient of the AI was significantly higher than that of the RSC and ACC (Fig. 4G; *P < 0.05).

## Discussion

The behavioural results of the present study are consistent with the findings of previous studies, showing that emotional contagion is increased in patients with AD^17^. Although Tg-11 mice had lower freezing levels in the fear memory test, consistent with previous studies^22^,^23^,^24^, indicating memory impairment, these mice showed considerably higher freezing levels in the OFC test compared to Wt-11 mice. Individual locomotor activity and freezing characteristics in fear conditioning did not affect the OFC freezing levels (Supplementary Fig. S1). This symptom was fully manifested when Tg mice were 11 months old, whereas Tg-7 mice showed a small increment in OFC freezing levels (Fig. 1). Thus, these findings indicate that elderly Tg mice with severe memory deficits express higher fear responses to the suffering of conspecifics than age-matched Wt mice.

To demonstrate the mechanism of heightened emotional contagion in Tg mice, changes in cross-correlation of LFP signals were investigated during OFC. The theta wave cross-correlation between the left AI and right BLA during OFC showed the highest increase in Tg-11 mice compared to Wt-11 mice, followed by that between the left ACC and right BLA (Fig. 2). Moreover, the correlation between the left AI and right BLA was significantly increased when Tg-11 mice observed suffering of others compared to the habituation session. Because the normalized theta power of the left AI in Tg-11 mice also increased during the conditioning session of OFC, the left AI may be the most relevant to elevated emotional contagion. Theta wave correlations in both Wt-11 and Tg-11 mice displayed general increases during the early phase of the conditioning session, but their paths diverged over time (Fig. 3). Whereas the initially increased pattern of Wt-11 mice decreased with time, Tg-11 mice maintained an elevated theta correlation throughout the session (Fig. 3B,C). This increased and sustained correlation in Tg-11 mice is consistent with the behavioural responses showing continuously high levels of freezing in OFC (Fig. 1D). Together, the prolonged increase in the correlation between left AI and right BLA is likely an important factor for heightened emotional contagion in Tg mice.

Furthermore, according to previous human brain network studies, the AI, ACC, and BLA regions belong to a SN^13^,^25^, and increased connectivity in this network is associated with emotional symptoms in patients with AD^14^. Previous studies have also reported that the connectivity between the AI and BLA, in particular, was increased by emotional stimuli such as an anxious context^26^ and chemicals related to emotional arousal^27^. Therefore, increased synchronized activity between the AI and BLA of Tg-11 mice during the emotional contagion task is consistent with the findings from human network studies. Additionally, the normalized power in delta range of the RSC, which corresponds to the human posterior cingulate cortex, a part of the default mode network (DMN), was lower in Tg-11 mice than in Wt mice (Supplementary Fig. S5). We can postulate that elderly Tg mice may also have low activation in the DMN-like regions, similar to AD patient studies^28^,^29^. As several studies have shown evidence for DMN in mice^30^,^31^, further studies of the relationship between DMN and SN in AD models are needed to understand network changes in patients with AD.

Interestingly, cross-correlation showed significant changes only in unilateral AI and BLA regions, consistent with previous human studies showing that the left AI is typically more active in empathic pain processing^32^,^33^, whereas the right BLA primarily processes negative emotion^34^. The lateralization of fear memory in the amygdala has also been reported in mice^35^,^36^. Although the laterality in empathic behaviour has been observed at the cortex level^37^, no studies have focused on the AI or BLA in mice. Further studies on the asymmetry of these areas may be helpful to understand the emotional circuits involved in AD.

Consistent with previous studies^22^,^38^, the Tg mice exhibited gradual accumulation of amyloid plaques with disease progression. All LFP recording regions showed a significant correlation between amyloid burden and the OFC freezing level. Among these regions, plaque burden in the AI, followed by BLA showed the highest correlation with the level of emotional contagion (Fig. 4). As previous studies of human patients have shown that alterations in brain connectivity are associated with amyloid deposit^39^, it is likely that highly accumulated amyloid plaques in the AI and BLA are related to the increased theta wave cross-correlation in the left AI and right BLA of Tg-11 mice (Fig. 2). Moreover, plaque burden in the hippocampus (HPC) which is related to the memory deficit in AD mice^22^ was significantly correlated with the fear memory deficit. Although plaque burden in the AI was also significantly correlated with the fear memory deficit, the correlation coefficient of HPC was significantly higher than that of AI (Supplementary Fig. S7). These results demonstrate that the AI may highly be involved in the emotional contagion rather than the fear memory function. Until recently, the AI in rodent AD models has not been demonstrated as a common area for quantifying the plaque burden or studying *in vivo* electrophysiology, although human studies have reported this amyloid burden^40^,^41^ and its functions in AD^42^,^43^. Thus, these results suggest the AI as an important target for studying emotional symptoms in AD models.

In conclusion, we demonstrated that Tg mice exhibit heightened emotional contagion in accordance with human studies. Furthermore, neural signal analysis of the SN-like regions revealed increased synchrony between the AI and BLA, where the accumulation of amyloid plaques correlates with the emotional contagion levels and is associated with this symptom. These findings provide a potential network connectivity mechanism for how and why patients with AD are easily influenced by the emotional state of caregivers.

## Materials and Methods

### Animals

Three-, seven-, and eleven-month-old male APPSWE/PS1E9 transgenic (Tg-3, Tg-7, and Tg-11, respectively) mice or their age-matched wild type (Wt-3, Wt-7, and Wt-11, respectively) littermates were used in the present study. C57BL/6 J mice were also used as demonstrators for observational fear conditioning. All mice were housed with a 12-h light/dark cycle (light 0900–2100, dark 2100–0900) and with *ad libitum* access to food and water. All behavioural tests were performed during the light phase. This study was approved by the Institutional Animal Care and Use Committee of KAIST, and animal care and handling were carried out according to their guidelines.

### Observational fear conditioning

OFC was performed as previously described 21. The apparatus for OFC comprised two identical chambers (each, 20 × 16 × 20 cm) with a transparent Plexiglas partition in the middle and a stainless steel grid floor (Med Associates, St. Albans, VT, USA). The mice (observer and demonstrator) were individually placed in separate apparatus chambers for 5 min (habituation session), and subsequently a foot shock (2 s, 1 mA) was delivered every 12 s for 4 min (conditioning session) to the demonstrator mouse. All behaviour sessions were video-recorded, and the freezing duration of the observer mouse was quantified after the test. Freezing was defined as the lack of movement (except for respiratory movements or sniffing) for longer than 1 s. One mouse was excluded from the data analysis as an outlier because the freezing value of the mouse was >2 standard deviation from the mean.

### Open field test

Spontaneous locomotor activity was measured using an open field test. The open field box was made of white plastic (40 × 40 × 40 cm). The mouse was placed on one side of the box, and activity was recorded for 30 min. The distance travelled every 5 min was analysed using EthoVision XT (Noldus, Wageningen, The Netherlands).

### Contextual fear conditioning

The mouse spent 5 min in the fear-conditioning chamber (26 × 26 × 24 cm) for habituation and subsequently received a foot shock (2 s, 0.7 mA) 4 times every 90 s. To assess contextual fear memory, the mouse was placed back into the same context after 24 h from the conditioning session. Fear response was video-recorded and measured after the test.

### Electrode implantation

Animals were anaesthetized through the intraperitoneal (i.p.) injection of 25 mg/kg Zoletil (1:1 mixture of tiletamine and zolazepam) and 12 mg/kg Rompun (xylazine). Electrode implantation was performed using a stereotaxic apparatus (Kopf Instruments, Tujunga, CA, USA). LFPs were obtained with custom-made tungsten electrodes (0.127 mm, 2 MΩ), which were positioned bilaterally in eight brain regions: ACC (AP + 1.8 mm, ML ± 0.3 mm, DV − 2.0 mm), AI (AP + 1.18 mm, ML ± 3.0 mm, DV − 3.9 mm), RSC (AP − 1.22 mm, ML ± 0.2 mm, DV − 1.0 mm), and BLA (AP − 1.6 mm, ML ± 3.35 − 3.45 mm, DV − 4.95 mm). A reference electrode was inserted on the skull over the cerebellum. These electrodes were fixed to the skull with dental acrylic cement. All electrode positions were histologically verified after the experiments.

### *In vivo* electrophysiology and analysis

Electrical signal recording combined with video monitoring were simultaneously conducted during the OFC test. Electrical activities were amplified (×1,200) and recorded, bandpass-filtered from 1 to 200 Hz, and digitized with a 1.6 kHz sampling rate using a digital system (Twin EEG software; Grass Technologies Inc., West Warwick, RI, USA). To calculate the cross-correlation coefficients during habituation and conditioning session, field potentials were filtered from 3 to 8 Hz (theta band range) and sampled at 10 s for habituation and 8 s for conditioning. For the conditioning period, foot shocks were administered every 12 s and lasted 2 s, and a total of twenty 8-s intervals were used for the analysis (excluding the 1 s from each end contaminated by the electrical shock stimuli). The relative normalized power at each individual frequency was presented in the manner of min-max normalization (xi − min(x)/max(x) − min(x)) at all frequencies of delta (1–3 Hz), theta (3–8 Hz), alpha (8–13 Hz), beta (14–30 Hz), and gamma (30–55 Hz). To calculate the matrix dissimilarity, Euclidean distance (d(i,j) = distance between matrix i and matrix j) was used as an index.


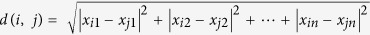


All data were analysed using MATLAB and pCLAMP 10 (Axon Instruments Inc., Foster City, CA, USA).

### Amyloid plaque quantification

To measure the deposition of amyloid plaques, thioflavin-S (Sigma-Aldrich, St Louis, MO, USA) staining was conducted. For the preparation of brain tissue slices, the mice were anaesthetized through i.p. injection using a mixture of Zoletil and Rompun and transcardially perfused with phosphate-buffered saline (PBS), followed by 4% paraformaldehyde (PFA). Extracted brains were incubated in 4% PFA at room temperature overnight. Cryosectioning was performed at 40-μm thickness, including the ACC, AI, RSC, and BLA. All slides were stained with thioflavin-S for 8 min at room temperature. The stained sections were scanned using a whole slide imaging system (Axio Scan.Z1, Zeiss, Jena, Germany) with a 10X objective lens. The percentage of amyloid plaque area in each region ((plaque area/area of each brain region) × 100) was analysed using ImageJ software (National Institutes of Health, Bethesda, Maryland, USA).

### Statistical analysis

Statistical analyses were conducted using SPSS software (SPSS Inc., Chicago, IL, USA) and Prism 5.0 (GraphPad Software Inc., San Diego, CA, USA). Two-way repeated ANOVA, two-way ANOVA and one-way ANOVA were used for behavioural analyses. Šidák and Bonferroni’s corrections were used for *post hoc* analysis. Spearman’s rank correlation was used to assess the correlation between amyloid burden and freezing behaviour. All other correlation data were analysed using Pearson’s method. To determine the significance of correlation coefficients in plaque burden, Steiger’s Z-test was used. In all cases, statistical significance was set at *P < 0.05. All data are shown as the mean ± the standard error of the mean (SEM).

## Additional Information

**How to cite this article**: Choi, J. and Jeong, Y. Elevated emotional contagion in a mouse model of Alzheimer’s disease is associated with increased synchronization in the insula and amygdala. *Sci. Rep.* **7**, 46262; doi: 10.1038/srep46262 (2017).

**Publisher's note:** Springer Nature remains neutral with regard to jurisdictional claims in published maps and institutional affiliations.

## Supplementary Material

Supplementary Information
