# Supplementary material for: Elevated emotional contagion in a mouse model of Alzheimer’s disease is associated with increased synchronization in the insula and amygdala
Source: Sci Rep. 2017 Apr 7;7:46262. doi: 10.1038/srep46262 (PMC5384199; doi:10.1038/srep46262)
Supplement: Supplementary Information [file srep46262-s1.pdf]

Elevated emotional contagion in a mouse model of Alzheimer's disease is associated with increased synchronization in the insula and amygdala

Jiye Choi, Yong Jeong*

Department of Bio and Brain Engineering, Korea Advanced Institute of Science and Technology (KAIST), Daejeon, 34141, Republic of Korea

KI Health Science and Technology, Korea Advanced Institute of Science and Technology (KAIST), Daejeon, 34141, Republic of Korea

Supplementary Information

7 Supplementary Figures + Legends

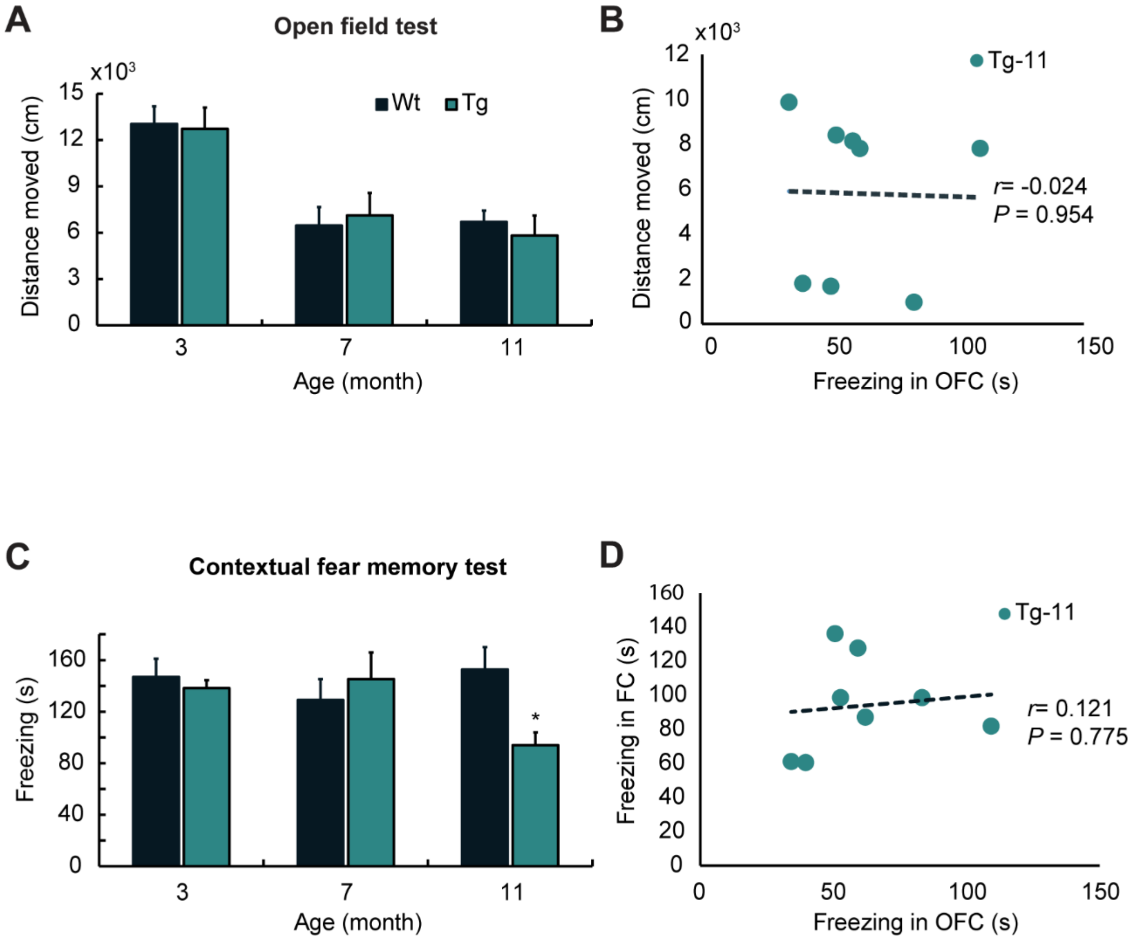

Supplementary Figure S1. Locomotor activity and fear memory levels of Tg mice and Wt littermates. (**A**) Total distance moved during 30 min in the open field box. (**B**) Relationship between the OFC freezing levels and the distance moved in the open field test. OFC freezing had no correlation with the locomotion in Tg-11 mice (r =– 0.024, $P = 0.954$, Pearson's correlation). (**C**) Tg-11 mice showed significantly less freezing duration in the contextual fear-conditioning (FC) test compared to the Wt-11 mice ($P < 0.05$ (genotype*age interaction), two-way ANOVA, *$P < 0.05$, Bonferroni's *post hoc* test). (**D**) Relationship between the OFC freezing and FC freezing levels in Tg-11 mice. There was no correlation between freezing levels in two different behaviour tests ($r = 0.121$, $P = 0.775$, Pearson's correlation). Data are presented as the mean ± SEM.

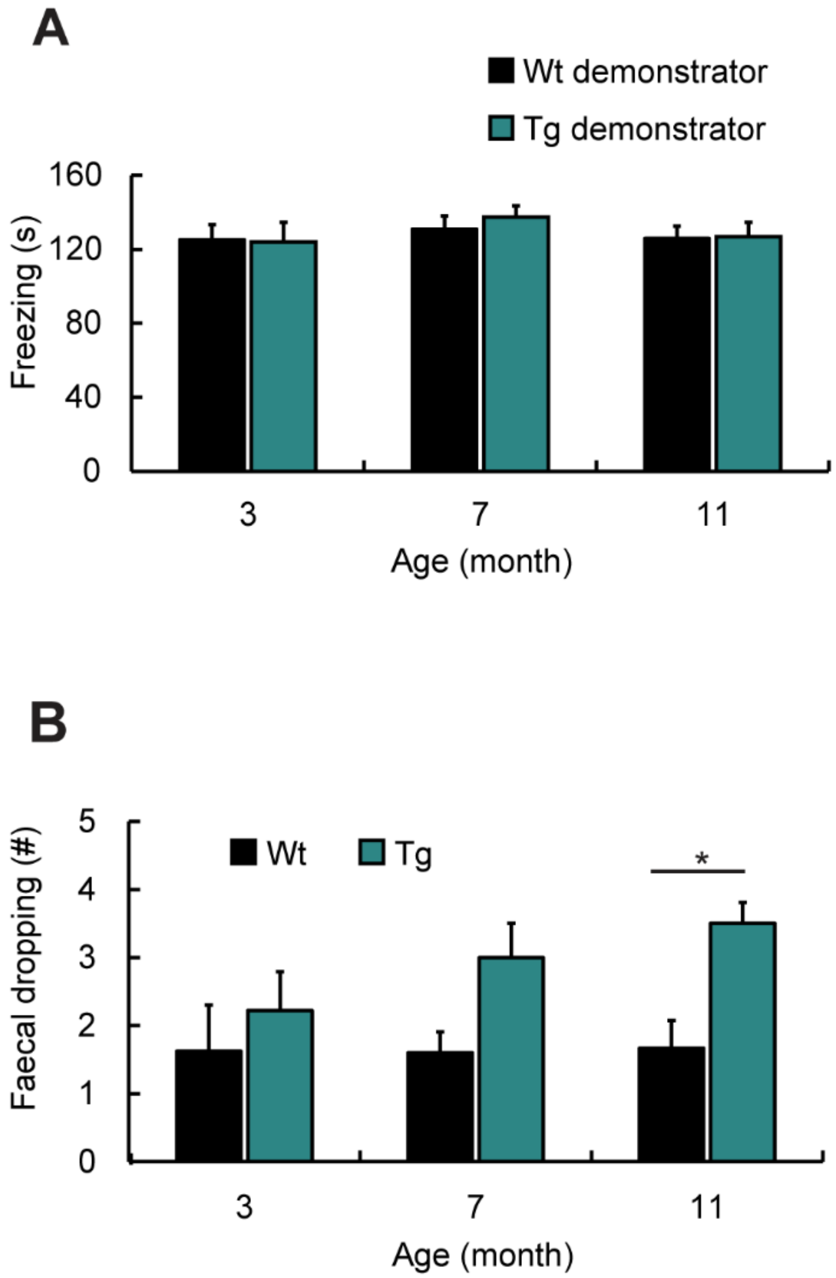

Supplementary Figure S2. Freezing levels in the demonstrators of each age group and the number of faecal droppings in the observers. (**A**) Freezing levels in the demonstrators of each group. There was no difference in the freezing levels in the demonstrators. (**B**) The number of faecal droppings, an indirect measure of fear, was significantly increased in Tg-11 mice compared to Wt-11 mice during the OFC test ($P < 0.01$ (genotype), two-way ANOVA, *$P < 0.05$, Bonferroni's *post hoc* test). Data are presented as the mean ± SEM.

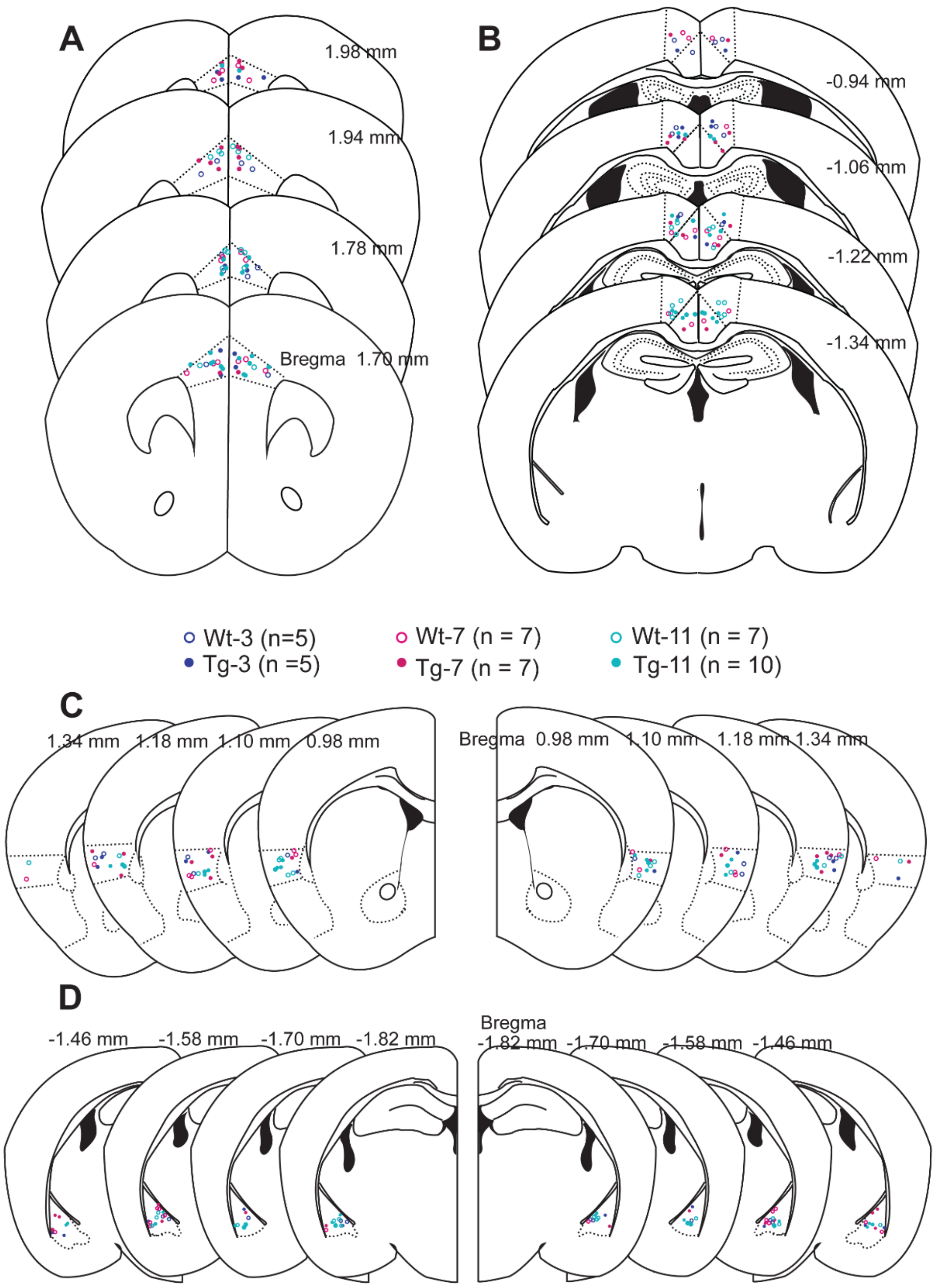

Supplementary Figure S3. Histologically verified locations of electrodes in four regions of interest. (**A-D**) Locations of electrodes used in LFP recording. Coronal sections from bregma of the ACC (**A**), RSC (**B**), AI (**C**), and BLA (**D**). Hollow circles and filled circles indicate the sites of electrodes for Wt mice and Tg mice, respectively.

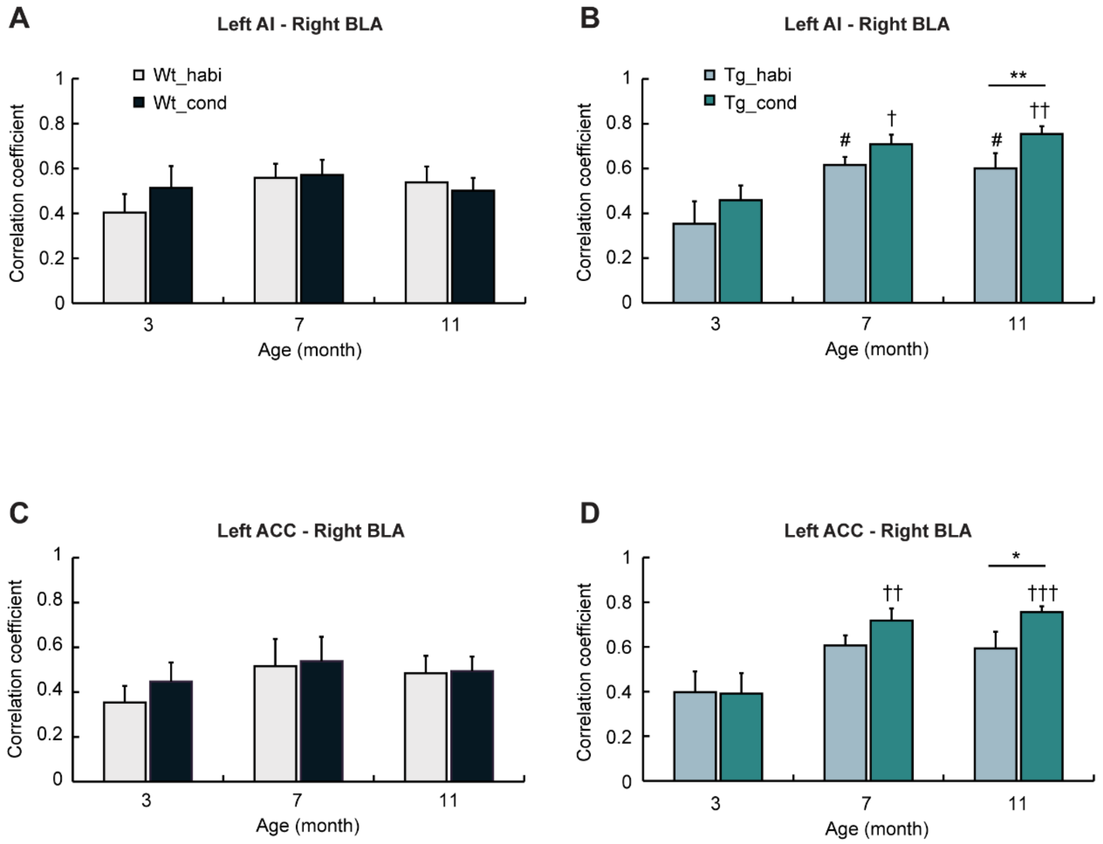

Supplementary Figure S4. The correlation changes in Tg mice as they got older. (**A-B**) The correlation between the left AI and right BLA. (**A**) There was no difference in Wt mice, according to the age factor. (**B**) The correlation between the left AI and right BLA in Tg mice was significantly increased as the mice got older (*P* < 0.01 (age), *P* < 0.005 (test), two-way repeated ANOVA, $^{\#}P < 0.05$ (compared to the Tg-3 habituation), $^{\dagger}P < 0.05$ (compared to the Tg-3 conditioning), Bonferroni's post hoc test). The correlation between two regions in Tg-11 was also significantly increased during the conditioning session compared to the habituation (**P < 0.01, Bonferroni's post hoc test). (**C-D**) The correlation between the left ACC and right BLA. (**C**) There was no difference in Wt mice, according to the age factor. (**D**) The correlation between the left ACC and right BLA in Tg mice was significantly increased as the mice got older (*P* < 0.01 (age), two-way repeated ANOVA, $^{\dagger\dagger}P < 0.01$, $^{\dagger\dagger\dagger}P < 0.05$ (compared to the Tg-3 conditioning), Bonferroni's post hoc test). The correlation between two regions in Tg-11 was also significantly increased during the conditioning session compared to the habituation (*P < 0.05, Bonferroni's post hoc test). habi: Habituation. cond: Conditioning. Data are presented as the mean ± SEM.

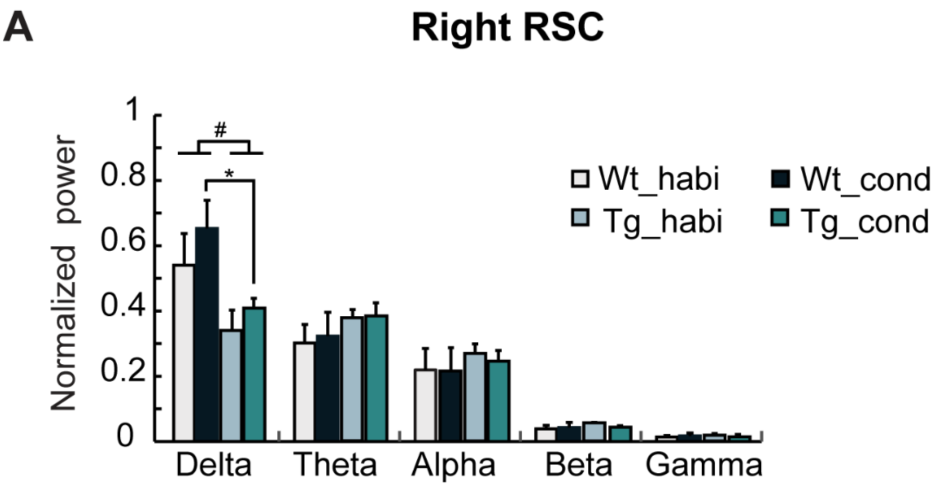

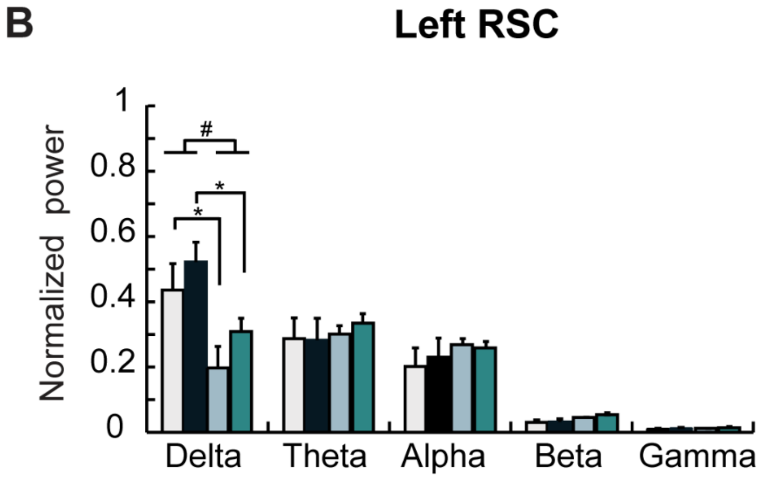

Supplementary Figure S5. Normalized power in the bilateral RSC regions of Wt-11 and Tg-11 mice. (A-B) Normalized power in the right (**A**) and left RSC (**B**). Normalized delta power of the right and left RSC in Tg-11 mice presented considerably lower levels than in Wt-11 mice ($^{\#}P < 0.05$ (genotype), two-way repeated ANOVA, $^{*}P < 0.05$, Bonferroni's *post hoc* test). habi: Habituation. cond: Conditioning. Data are presented as the mean ± SEM.

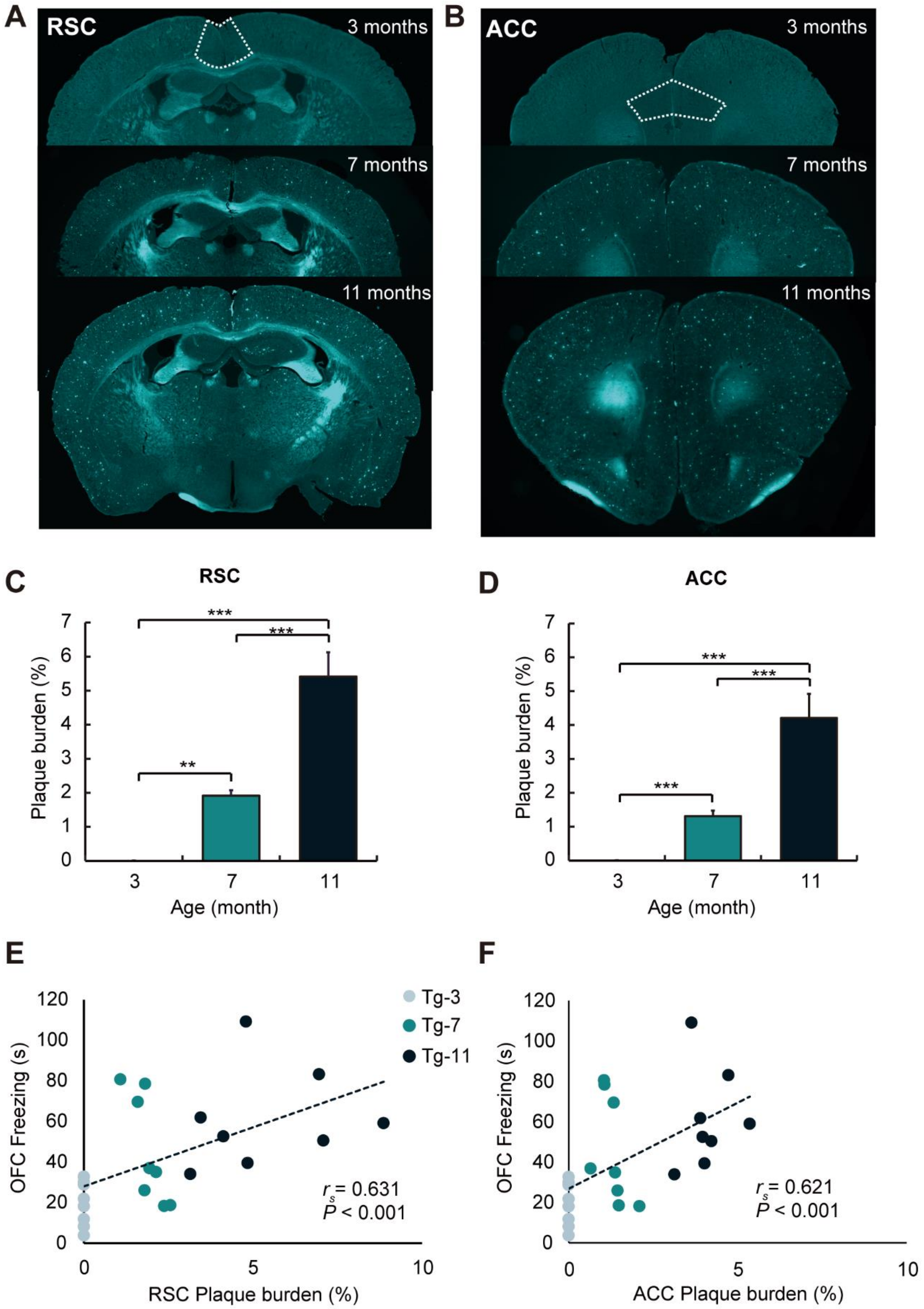

A
RSC
3 months
7 months
11 months
B
ACC
3 months
7 months
11 months
C
RSC
Plaque burden (%)
Age (month)
**
***
***
D
ACC
Plaque burden (%)
Age (month)
***
***
***
E
OFC Freezing (s)
RSC Plaque burden (%)
Tg-3
Tg-7
Tg-11
r_s = 0.631
P < 0.001
F
OFC Freezing (s)
ACC Plaque burden (%)
r_s = 0.621
P < 0.001

Supplementary Figure S6. Increase in amyloid plaque burden in the RSC and ACC of Tg mice has a correlation with freezing levels in OFC. (A, B) Examples of amyloid plaque deposition in the RSC (**A**) and ACC (**B**) of Tg-3, Tg-7, and Tg-11. (**C, D**) Average plaque burden of the RSC (**C**) and ACC (**D**) were quantified in Tg-3, Tg-7, and Tg-11. All three age groups showed significant differences in plaque burden in the RSC (**C**; $P < 0.0001$ (age), one-way ANOVA) and ACC (**D**; $P < 0.0001$ (age), one-way ANOVA). Bonferroni's correction was used for the *post hoc* analysis. $^{*}P < 0.05$, $^{***}P < 0.001$. (**E-F**) Plaque burden both in the RSC (**E**; $r_s = 0.631$, $P < 0.001$) and ACC (**F**; $r_s = 0.621$, $P < 0.001$) showed significant correlations with the freezing levels in OFC. Spearman's correlation was used for the significance test. Data are presented as the mean ± SEM.

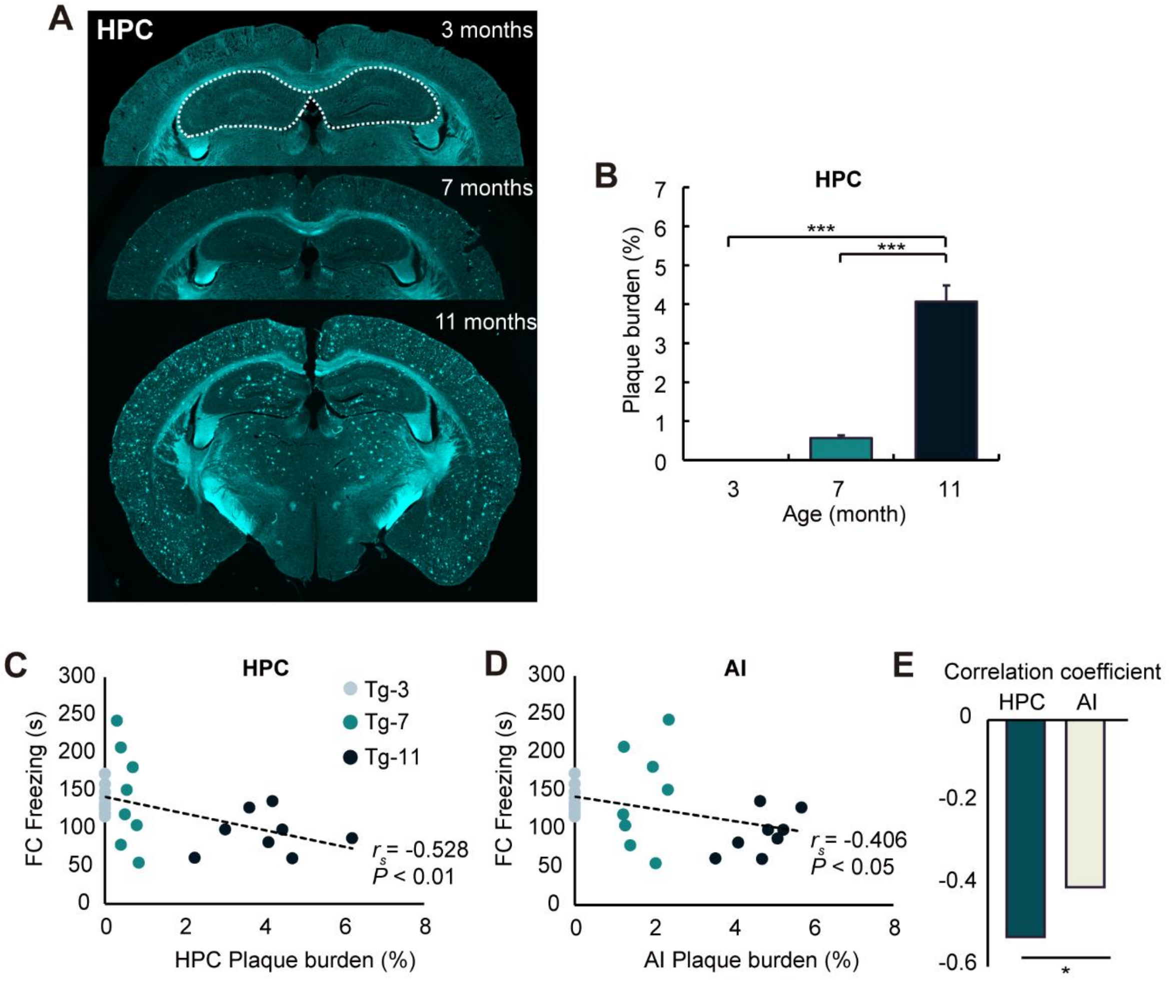

Supplementary Figure S7. Amyloid plaque burden in the hippocampus of Tg mice has a correlation with freezing levels in FC. (**A**) Example of amyloid plaque deposition in the hippocampus (HPC). (**B**) Average plaque burden of the HPC was quantified in Tg-3, Tg-7, and Tg-11. There was no difference between Tg-3 and Tg-7 mice, but Tg-11 mice showed a significantly higher plaque burden compared with Tg-3 or Tg-7 animals ($P < 0.0001$ (age), one-way ANOVA, ***$P < 0.001$, Bonferroni's *post hoc* test). (**C-D**) Plaque burden both in the HPC (**C**; $r_s = -0.528$, $P < 0.01$) and AI (**D**; $r_s = -0.406$, $P < 0.05$) showed significant correlations with the freezing levels in FC. Spearman's correlation was used for the significance test. (**E**) The correlation coefficient of the HPC was significantly higher than that of the AI (*$P < 0.05$, Steiger's z-test). Data are presented as the mean ± SEM.
